# Atypical Disseminated Herpes Zoster With Rapid Spread in an Older Adult

**DOI:** 10.7759/cureus.111509

**Published:** 2026-06-25

**Authors:** Gokul Paidi, Gifty Thomas, Pushpa Yadav, Jashvini Amirthalingam, Gandhy Montalvo Rosales, Shivangi Yadav

**Affiliations:** 1 Family Medicine, Community Healthcare Network, New York, USA

**Keywords:** disseminated herpes zoster, elderly patient, genital zoster, sacral zoster, scrotal involvement, varicella zoster virus

## Abstract

Disseminated herpes zoster is an uncommon and severe presentation of varicella zoster virus reactivation that may involve widespread cutaneous lesions and atypical distributions. Although it is more frequently observed in immunocompromised individuals, it can also occur in immunocompetent patients, particularly in older adults, and may present with generalized eruptions that resemble varicella. We report a case of atypical disseminated herpes zoster with involvement of the gluteal region, genitalia, and bilateral lower extremities in an immunocompetent patient, highlighting an unusual clinical presentation and the importance of early recognition.

## Introduction

Herpes zoster results from reactivation of latent varicella zoster virus within sensory ganglia and typically presents as a unilateral dermatomal vesicular eruption. Disseminated herpes zoster is less common and defined by widespread cutaneous lesions beyond adjacent dermatomes, with or without visceral involvement [1,2]. It most often occurs in immunocompromised patients, although advanced age alone may increase risk due to declining cell-mediated immunity [1-3].

This case is notable for rapid progression from a localized gluteal eruption to bilateral lower extremity and genital involvement within 48 hours, requiring intravenous antiviral therapy in an immunocompetent patient without identifiable risk factors, followed by persistent genitourinary symptoms. Age-related decline in cell-mediated immunity may contribute to rapid dissemination even in otherwise immunocompetent older adults. The key learning point is that rapid dissemination with genital involvement and prolonged sequelae can occur in the absence of overt immunosuppression.

## Case presentation

**Figure 1:**
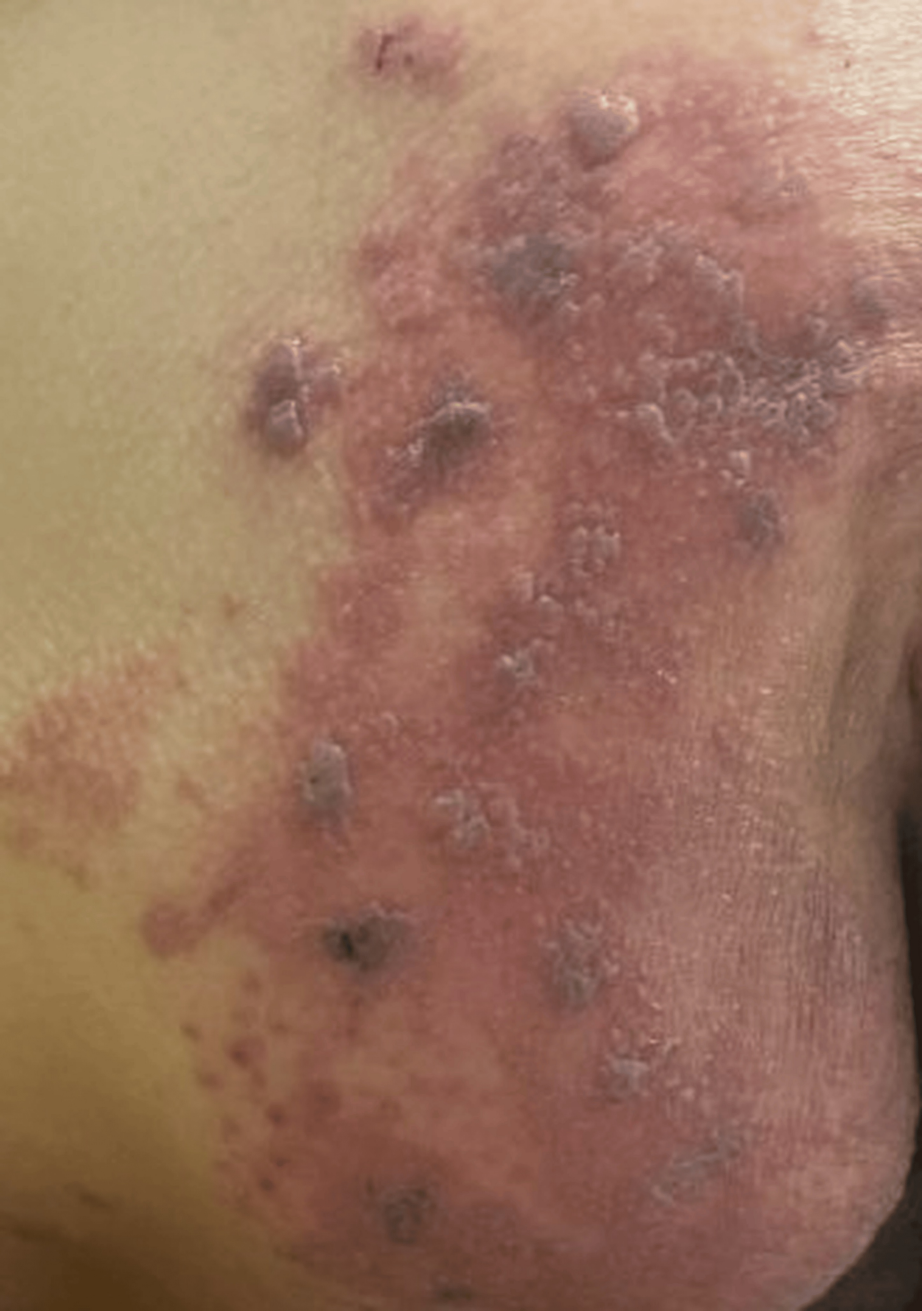
Initial lesions on left gluteal region with scattered dark hemorrhagic crusted macules and papules.

A 78-year-old man presented to the ED after noticing a rash involving the buttocks earlier that day. His medical history included hypertension, hyperlipidemia, coronary artery disease, chronic kidney disease, carotid artery occlusion, arthritis, and prior paroxysmal atrial tachycardia. He denied itchiness or burning and described the area as uncomfortable. He otherwise felt well and denied abdominal pain, nausea, vomiting, fever, chills, rectal pain, urinary symptoms, or drainage. He is not on any immunosuppressive medications at home. He is hemodynamically stable. Based on the clinical impression, the differential diagnosis included herpes zoster, with lower suspicion for cellulitis, abscess, or tinea infection. He was prescribed Valtrex 1 g BID and discharged with follow-up as needed. The initial lesion is shown in Figure 1.

Two days later, he returned to the emergency department because of worsening rash involving the left gluteal region with extension to both lower extremities. He described the rash as tender to touch but denied pruritus. He also denied fever, chills, respiratory symptoms, gastrointestinal symptoms, dysuria, penile discharge, or recent sexual activity.

**Figure 2:**
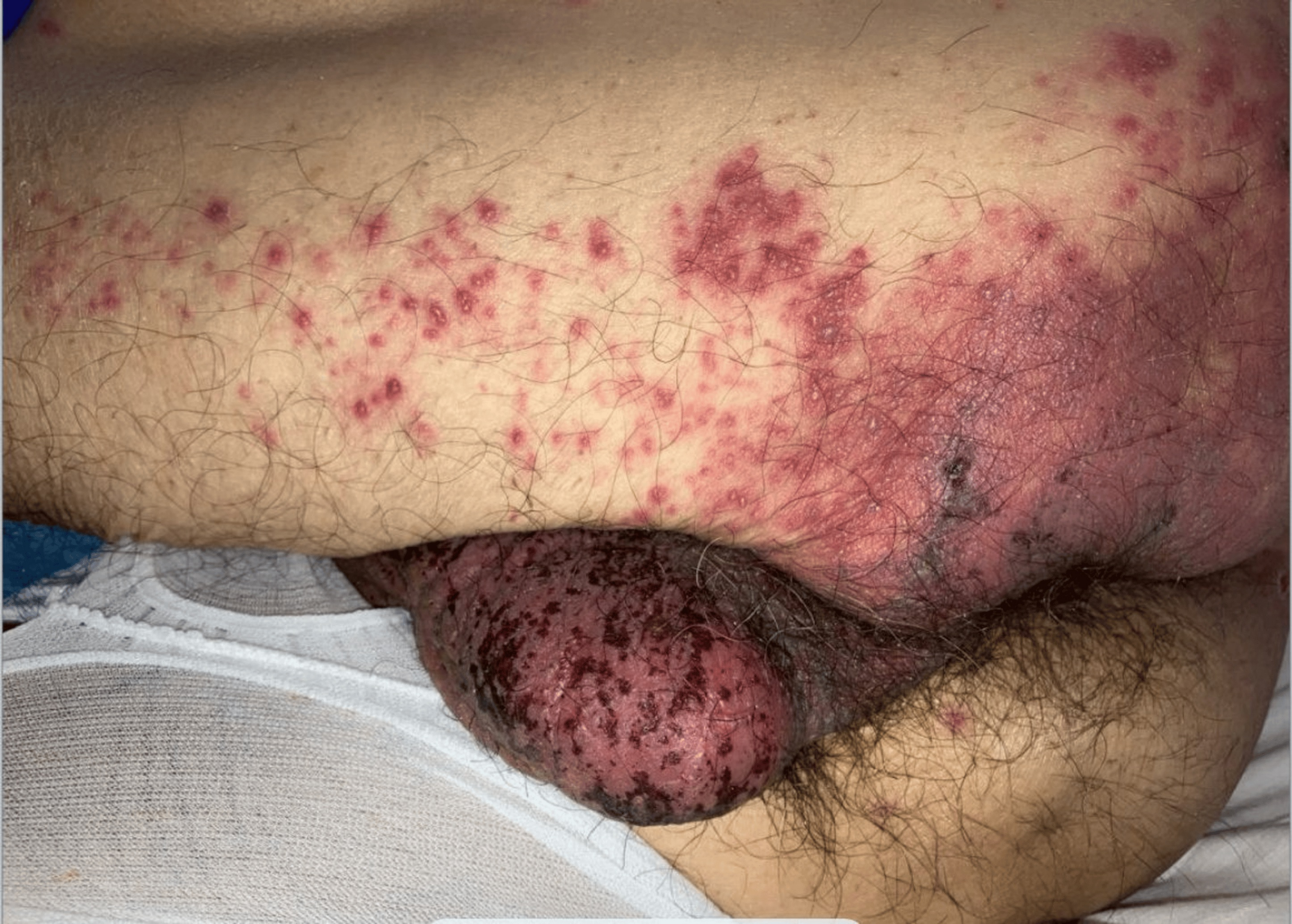
Left testicle with dark violaceous, crusted, hemorrhagic lesions; severe scrotal involvement plus scattered erythematous vesiculopapular lesions extending across the medial thigh/groin.

On presentation, he was afebrile and hemodynamically stable. Physical examination revealed a vesicular and maculopapular eruption in various stages of evolution involving the left gluteal region, with additional papular lesions on both lower extremities. Multiple papulovesicular lesions were also present on the penis and left hemiscrotum. Clinical photographs demonstrated as well as marked scrotal and perineal inflammation (Figure 2).

Laboratory evaluation was notable for leukopenia (WBC: 2.8), anemia (hemoglobin: 10.3 g/dL), mild acute kidney injury (creatinine: 1.35 mg/dL), iron deficiency, and vitamin B12 deficiency. Electrocardiography showed sinus rhythm with premature atrial complexes. Given the bilateral distribution and multidermatomal involvement, disseminated herpes zoster was suspected. Infectious disease recommended treatment with intravenous acyclovir 5 mg/kg q8h, and the patient was admitted under isolation precautions. The sexually transmitted infection (STI) panel was negative, and varicella zoster virus (VZV) PCR returned positive. During hospitalization, renal function improved with supportive care, with creatinine decreasing to 0.96 mg/dL. The patient’s bicytopenia is suspected to be secondary to vitamin B12 deficiency. He was initiated on vitamin B12 and ferrous sulfate supplementation. Cardiology was consulted because of his history of paroxysmal atrial tachycardia, but no acute intervention was required.

After three days of intravenous acyclovir, the lesions began to crust and demonstrated clinical improvement. He was transitioned to oral valacyclovir 1 g BID for five days to complete therapy and was discharged home in stable condition. At discharge, residual lesions remained on the left buttock and scrotum with scattered lesions on the extremities, although most had crusted. No facial, ophthalmic, or visceral involvement was identified.

Follow-up presentations

He returned about 12 days later with two to three days of left testicular pain and burning with urination. He did not have fever, penile discharge, nausea, vomiting, blood in the urine, or flank pain. On exam, he was uncomfortable. The left testicle was red and tender. There was tenderness of the penis and testicle, but no rash, swelling, penile discharge, or skin redness of the penis.

Urinalysis showed large blood, many red blood cells, and 5 to 10 white blood cells per high-power field. CT pelvis with IV contrast showed diffuse bladder wall thickening, mild enhancement of the partially seen right proximal ureter, and mild prostatomegaly with intravesical protrusion. No definite penile abnormality was seen. Scrotal ultrasound showed mildly heterogeneous echogenicity of both testicles, which was nonspecific and may reflect sequelae of epididymo-orchitis. There was no testicular torsion or testicular mass. Small bilateral hydroceles with internal debris and bilateral scrotal calcifications, likely related to prior inflammation, were also noted. Differential diagnosis included cystitis, urethritis, epididymo-orchitis, and postherpetic neuralgia. Urine culture and STI panel were negative. He appeared overall well, was treated with pain control, and was discharged.

## Discussion

This case highlights the rapid progression of an initially localized sacral herpes zoster eruption to disseminated cutaneous disease in an older adult without a clearly identifiable major immunocompromising condition [4-6]. Although the rash initially appeared confined to the left gluteal region, it progressed within 48 hours to involve the penis, scrotum, and bilateral lower extremities, prompting hospitalization and treatment with intravenous acyclovir. A notable feature of this case was the relatively mild initial presentation. Despite extensive skin involvement, the patient reported only discomfort and tenderness, without fever, pruritus, or other systemic symptoms. This subtle presentation may contribute to delayed recognition of disease progression, particularly when the eruption initially appears localized [4-7].

The distribution of lesions was also unusual. While sacral herpes zoster commonly involves the buttock and genital region, the presence of bilateral lower extremity lesions suggested dissemination beyond a single dermatome [5-8]. Fortunately, there was no evidence of ophthalmic, neurologic, or visceral involvement. The patient responded well to intravenous acyclovir followed by oral valacyclovir, with lesion crusting and clinical improvement within several days [8-10]. His hospital course was complicated by a transient acute kidney injury that resolved with supportive care, highlighting the importance of monitoring renal function during antiviral therapy.

An additional learning point is the persistence of genitourinary symptoms after resolution of the rash. In the weeks following discharge, the patient returned with penile pain, testicular pain, and dysuria despite the absence of recurrent skin lesions. Imaging findings were nonspecific and showed no acute surgical pathology. Given the prior genital involvement, these symptoms were felt to be related to ongoing inflammatory or neuropathic sequelae of sacral varicella zoster virus reactivation. This case illustrates that clinical recovery may extend beyond cutaneous healing and that persistent genitourinary symptoms can occur after sacral and genital herpes zoster [10-13].

## Conclusions

This case describes disseminated herpes zoster in a 78-year-old man whose rash rapidly progressed from the left gluteal region to involve the genitalia and bilateral lower extremities within two days. He improved with intravenous acyclovir followed by oral valacyclovir but later developed persistent penile and testicular pain with dysuria after the cutaneous lesions had resolved. This case highlights that disseminated zoster can occur in older adults without obvious immunosuppression, that sacral and genital involvement may mimic other acute genitourinary conditions, and that prompt antiviral therapy is warranted when lesions become multidermatomal or widespread. Persistent genitourinary symptoms may represent postherpetic or inflammatory sequelae of sacral varicella zoster virus reactivation and may require ongoing follow-up.
